# Carbon ceramic microelectrodes modified with buckyballs for simultaneous determination of redox-active biomolecules

**DOI:** 10.1039/c7ra09603h

**Published:** 2018-02-06

**Authors:** Z. Omara Shastan, Hashwin V. S. Ganesh, Meissam Noroozifar, Kagan Kerman

**Affiliations:** Department of Chemistry, University of Sistan and Baluchestan P. O. Box 98135-674 Zahedan Iran mnoroozifar@chem.usb.ac.ir; Department of Physical and Environmental Sciences, University of Toronto Scarborough 1265 Military Trail Toronto Ontario M1C 1A4 Canada kagan.kerman@utoronto.ca

## Abstract

In this report, simultaneous electrochemical determination of ascorbic acid (AA), dopamine (DA), uric acid (UA) and tryptophan (Trp) was achieved using buckyball-modified carbon ceramic microelectrodes (CCMEs). A concentration-dependent increase in anodic peak current signals was observed in comparison with those obtained at bare CCMEs. The optimal pH for simultaneous determination of a quaternary mixture of AA–DA–UA–Trp was determined to be pH 4. The peak separations for the mixture containing AA–DA–UA–Trp were well-defined at the scan rate of 50 mV s^−1^. The catalytic peak current obtained was linearly dependent on the AA, DA, UA and Trp concentrations in the range of 6.0–600, 6.0–600, 6.0–600 and 4.0–440 μM, respectively. The detection limits for AA, DA, UA and Trp were also determined to be 1.64, 0.82, 0.36 and 1.22 μM, respectively. The analytical performance of this sensor has also been challenged for simultaneous electrochemical detection of AA, DA, UA and Trp in real samples.

## Abstract 2

In this report, simultaneous electrochemical determination of ascorbic acid (AA), dopamine (DA), uric acid (UA) and tryptophan (Trp) was achieved using buckyball-modified carbon ceramic microelectrodes (CCMEs).

## Graphical abstract

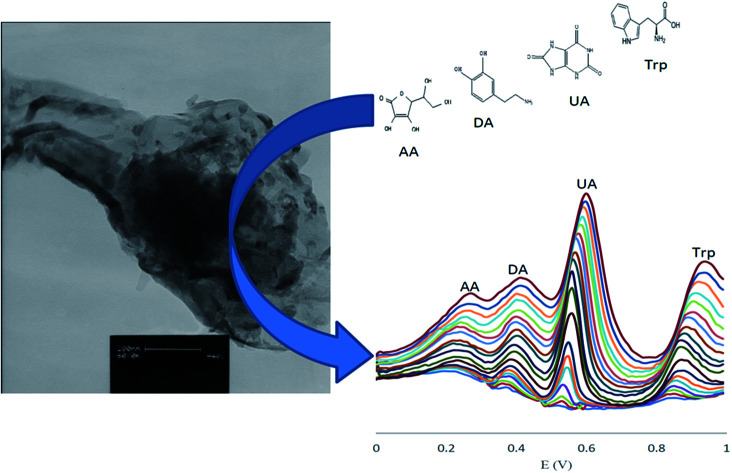

## 1. Introduction

Development of electrochemical sensors for simultaneous determination of multiple analytes is a rapidly growing area of research. Simultaneous analytical detection of physiologically important analytes such as dopamine (DA), ascorbic acid (AA), uric acid (UA) and tryptophan (Trp) has profound implications for disease diagnostics and characterization of disease pathology. For instance, DA, a catecholamine, is an important neurotransmitter that plays a vital role in the brain's reward and pleasure pathways.^1^ DA is also implicated in diseases such as Parkinson's disease (PD), a neurodegenerative disorder characterized by the degradation of dopaminergic neurons in the substantia nigra (SNR) region of the brain.^2^ Similarly, detection of UA, a primary end product of purine metabolism, is highly relevant for conditions such as gout, wherein, excess UA in gout patients results in the formation of crystals in bone joints.^3^ On the other hand, analytes such as AA and Trp are implicated in functions that are fundamental to the human metabolism.^4,5^ For instance, maintaining adequate levels of AA, commonly referred to as vitamin C, is important for preventing diseases such as scurvy and neurodegenerative diseases such as Alzheimer's disease (AD).^6,7^ Therefore, detecting and tracking levels of AA has diagnostic relevance for the aforementioned diseases.

Simultaneous determination of DA, AA, UA and Trp using non-modified electrodes (such as metal, carbon paste) is not viable because the anodic peak potentials of analytes overlap and the adsorption of oxidized analytes on the electrode surface, result in surface fouling.^8–10^ It is, therefore, important to develop specially modified electrodes for the simple, effective and simultaneous determination of analytes. Various groups have reported interesting electrode modifications using nano-composites,^11–13^ nanoparticles,^14–16^ polymer films,^17–23^ carbon nanotubes (CNTs),^24,25^ metal oxide^26,27^ and self-assembled monolayers^28^ for the simultaneous determination of AA, DA, UA and Trp. Recently, only a few modified electrodes have been reported for the simultaneous determination of AA, DA, UA and Trp. For example, Wang *et al.*^29^ have used multi-walled carbon nanotube and reduced graphene oxide hybrid functionalized by poly(amido-amine) and Au nanoparticles for simultaneous determination of AA, DA and UA. Yan *et al.*^30^ have reported a glassy carbon electrode modified with the [Ni(phen)_2_]^2+^ complex and single-walled carbon nanotubes for the simultaneous determination of AA, DA and UA. Zhao *et al.*^31^ have synthesized a hierarchical structure of Pt–Cu alloy and used it for the simultaneous determination of AA, DA and UA. Yang and Li^32^ have developed a modified glassy carbon electrode (MGCE) hexadecyltrimethylammoniumbromide functionalized graphene oxide/multi-walled CNTs for simultaneous determination of AA, DA and UA. Prathap and Srivastava^33^ have developed a MGCE with transition metal ion-exchanged mesoporous polyaniline for simultaneous determination of AA, DA, UA and Trp. Kaur *et al.*^34^ used a MGCE with silver nanoparticle-decorated reduced graphene oxide composite for simultaneous determination of AA, DA, UA and Trp. Yang *et al.*^35^ have reported a MGCE with carbon-supported NiCoO_2_ nanoparticles for the simultaneous determination of AA, UA, Trp and adenine.

Previously, we developed a MGCE with iron ion-doped natrolite zeolite multi-walled CNTs as a very stable, sensitive, selective and stable inorganic modifier for simultaneous determination of AA, DA, UA and Trp.^36^ In this work, we have developed a sol–gel (SG) carbon ceramic electrode (CCE) fabricated using a facile method involving microwave irradiation (MW) as first reported by Abbaspour *et al.*,^37^ for the simultaneous detection of AA, DA, UA and Trp. The use of MW radiation for driving chemical reactions has been reported for synthesis of various organic^38^ and inorganic materials^39^ and polymers.^40^ MW has many advantages over conventional heating process such as offering increased efficiency, improved production speed and decreased production costs. Moreover, the use of MW offers better control over the heating process, as MW can be turned on and off instantly. In this study, MW irradiation was successfully used to produce a SGCCE, with a fabrication time of 10 min compared to conventional methods that typically take 48 h.^41,42^ Using this modified CCE, we were able to successfully detect AA, DA, UA and Trp in the μM concentration range and achieved a detection limit of 1.64 μM, 0.82 μM, 0.36 μM and 1.22 μM, respectively. Finally, the modified electrode was used for simultaneous determination of AA, DA, UA and Trp in real samples.

## 2. Experimental

### 2.1 Reagents and materials and preparation of practical solutions

Methyltrimethoxysilane (MTMOS) was purchased from Fluka (Oakville, ON) and used without further purification. Buckyball (with purity 98%) (BB), high purity graphite powder, AA, DA, UA and Trp were obtained from Sigma-Aldrich (Oakville, ON). All other reagents were purchased from Merck Company (Kenilworth, NJ). Phosphate buffer solutions (PBS) were prepared from H_3_PO_4_ (0.1 M), and pH range was adjusted to 2.0–7.0 with 0.1 M H_3_PO_4_ and NaOH. All solutions were prepared with deionized distilled water (DDW). All experiments were performed in compliance with the relevant laws and institutional guidelines. In addition, we include a statement that informed consent was obtained for any experimentation with human subjects. Human urine and blood serum samples were obtained from the Sina Clinical Laboratory, Zahedan, Iran. The samples were acquired from human volunteers by a medical examiner. The samples were frozen at −20 °C immediately after collection and were shipped after retrieval at the earliest by the medical examiner's office to prevent loss of analytes by degradation processes. The blood serum and urine samples were diluted 10 and 5 times with PBS (pH 4.0) to produce a solution of AA, DA, UA and Trp with a concentration of 100 μM. DPV, in conjunction with standard addition technique, was used for the determination of the AA, DA, UA and Trp contents of the samples.

### 2.2 Instrumentation

Electrochemical measurements were performed with a SAMA-500 electroanalyzer (SAMA Research Center, Iran) and an Autolab electrochemical analyzer (Metrohm Autolab B. V., Utrecht, The Netherlands). All electrochemical experiments were carried out in a conventional three-electrode cell at room temperature. A platinum electrode and a silver/silver chloride electrode (Ag/AgCl) were used as the counter and reference electrodes, respectively. A modified CCE with BB was used as working electrode. A Metrohm pH meter, model 744 was used for pH measurements. TEM images were taken using a Philips CM120 transmission electron microscopy with 2.50 Å resolution.

### 2.3 Preparation of CCE modified with BB

MTMOS (33 μL), CH_3_OH (50 μL), and 10 μL HCl (4 mol L^−1^) were mixed and stirred for 2 min until a homogeneous gel solution appeared. Then, 125 mg graphite powder and 40 mg BB were added, and the resulting mixture was sonicated for an additional 10 min, then the mixture was heated in a microwave at 720 watts at 150 °C for 10 min, and was powdered in a vibrator for 20 min. The mixture was packed in a micro-hematocrit tube and a copper wire was inserted into the CCE as an electrical contact. This modified electrode was denoted as CCE-BB. The bare carbon ceramic electrode was prepared using the same procedure without the addition of BB. The electrode was then placed in 0.1 mol L^−1^ NaOH and electrode potential was cycled between 0.0 and 1.0 V (*vs.* Ag/AgCl) at a scan rate of 50 mV s^−1^ for 15 cycles in a cyclic voltammetry regime until a stable voltammogram was obtained. This modified electrode was denoted as CCE. When not in use, the modified electrode was stored in DDW.

## 3. Result and discussion

### 3.1 Characterization

**Fig. 1:**
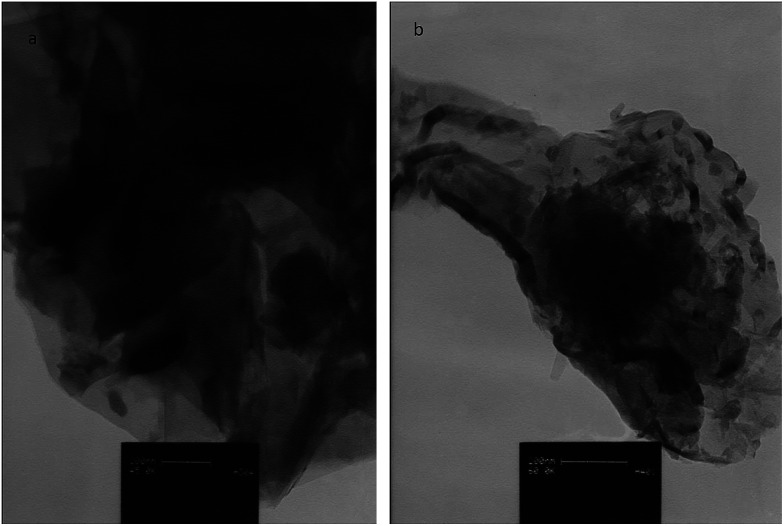
TEM images of (a) CCME and (b) MCCME-BB. The scale bar indicates 200 nm.

Particle morphology for the CCME and MCCME-BB samples are shown by TEM micrographs in Fig. 1. The MCCME sol–gel derived BB powder possessed a more equi-axed morphology than the CCME; sizes were mostly 40–80 nm with a maximum of ∼150 nm.

### 3.2 Voltammetric characterization of CCME and MCCME-BB

**Fig. 2:**
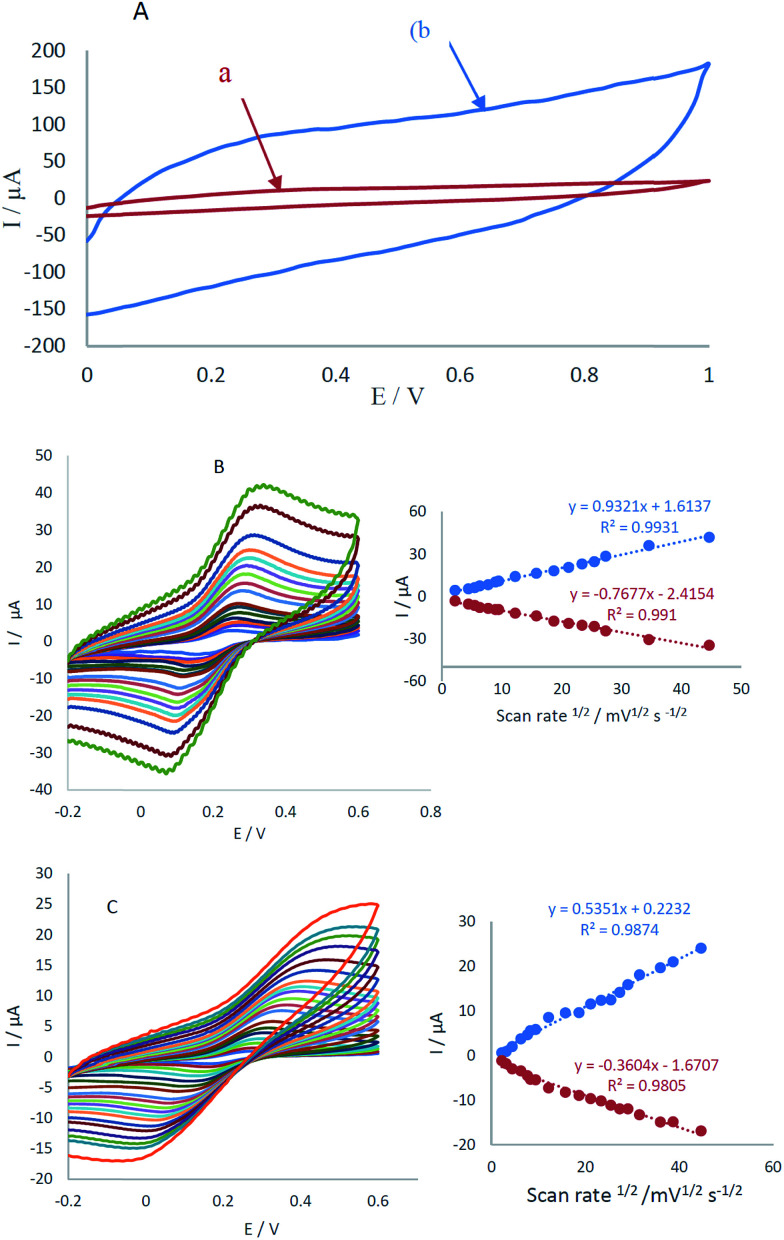
(A) Electrochemical behavior of (a) CCME and (b) MCCME-BB in PBS (pH 4.0) at a scan rate of 50 mV s^−1^ and CVs of (B) MCCME-BB and (C) CCME of 10 mM [Fe(CN)_6_]^3−/4−^ prepared in 0.1 M KCl at different scan rates, (insets; plots of *I vs. v*^1/2^ of B and C, respectively).

The electrochemical characterization of CCME and MCCME-BB in potential range 0.0–1 V *vs.* Ag/AgCl is shown in Fig. 2A. No detectable redox peaks were noticed (see Fig. 2A, curves a and b), but the charge transfer current of MCCME-BB was measured to be five times more than CCME. Electrochemical measurements of the CCME and MCCME-BB were analyzed for the anodic peak current (*I*_pa_) of the respective cyclic voltammograms obtained in the presence of 1.0 mM of [Fe(CN)_6_]^3−/4−^ in 0.1 M KCl supporting electrolyte (Fig. 2B and C). All assays were performed using cyclic voltammetry between potentials of −0.2 to 0.6 V as a probe at different scan rates. For a reversible process, the Randles–Sevcik equation can be used.^43^

1 *I*_pa_ = 2.69 × 10^5^*n*^3/2^*AC*_0_*D*_R_^1/2^*v*^1/2^

where *I*_pa_ refers to the anodic peak current, *n* is the electron transfer number, *A* is the surface area of the electrode, *D*_R_ is the diffusion coefficient, *C*_0_ is the concentration of [Fe(CN)_6_]^3−/4−^ and *v* is the scan rate. For 1 mM [Fe(CN)_6_]^3−/4−^ in the 0.1 M KCl electrolyte, *n* = 1 and *D*_R_ = 7.6 × 10^−6^ cm s^−1^; the microscopic areas were calculated from the slope of the *I*_pa_–*v*^1/2^ relation. For CCME and MCCME-BB, the electrode surface was found to be 0.25 and 0.46 cm^2^, respectively.

**Fig. 3:**
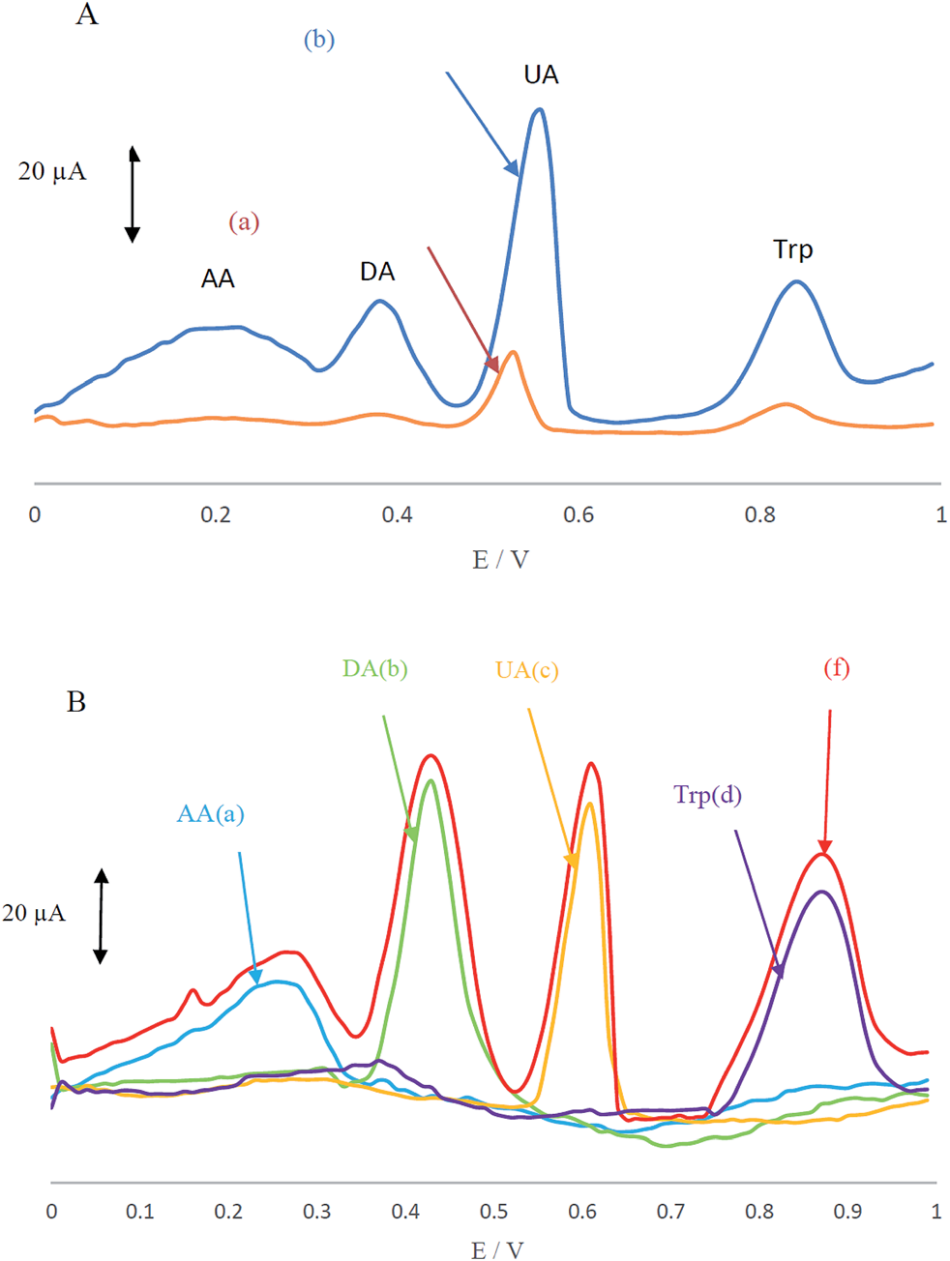
(A) Differential pulse voltammograms of (a) CCME and (b) MCCME-BB in PBS (pH 4.0) containing AA (400 μM), DA (200 μM), UA (200 μM) and Trp (200 μM) and (B) differential pulse voltammograms of MCCME-BB for individual solutions of (a) AA, (b) DA, (c) UA, (d) Trp and (f) a mixture of AA, DA, UA, Trp.

Electrochemical behaviour of the analyte mixture containing AA, DA, UA and Trp in 0.1 M PBS at pH 4 was carefully investigated at the surfaces of CCME and MCCME-BB using differential pulse voltammetry (DPV). CCME electrode showed two weak and broad oxidation peaks for UA and Trp at 0.53 and 0.83 V, suggesting slow electron transfer kinetics and without any oxidation peaks for AA and DA (see Fig. 3A). In contrast, the MCCME-BB showed four well-defined and sharp peaks for AA, DA, UA and Trp at 0.21, 0.38, 0.55 and 0.85 V, respectively.

As shown in Fig. 3A, the oxidation peaks of AA, DA, UA and Trp were separated with a considerable enhancement in the anodic peak current of AA, DA, UA and Trp by using the MCCME-BB. These results indicated that the sensitivity of well-separated voltammetric signals (four anodic peak current signals) corresponded to their oxidation and were found to be significant enough to apply for sensitive and simultaneous determination of AA, DA, UA and Trp in mixtures. The potential peak separation for AA–DA, DA–UA and UA–Trp are 0.2 V, 0.07 V and 0.3 V, respectively, which was suitable for the simultaneous determination of four compounds. Fig. 3B display the DPVs of individual solutions of AA, DA, UA and Trp and a mixture of AA, DA, UA and Trp in the potential range 0.0–1.0 V. As shown in Fig. 3B, the peak potentials of AA, DA, UA and Trp were constant in both experimental conditions.

### 3.3 Effect of pH on the oxidation of AA, DA, UA and Trp

**Fig. 4:**
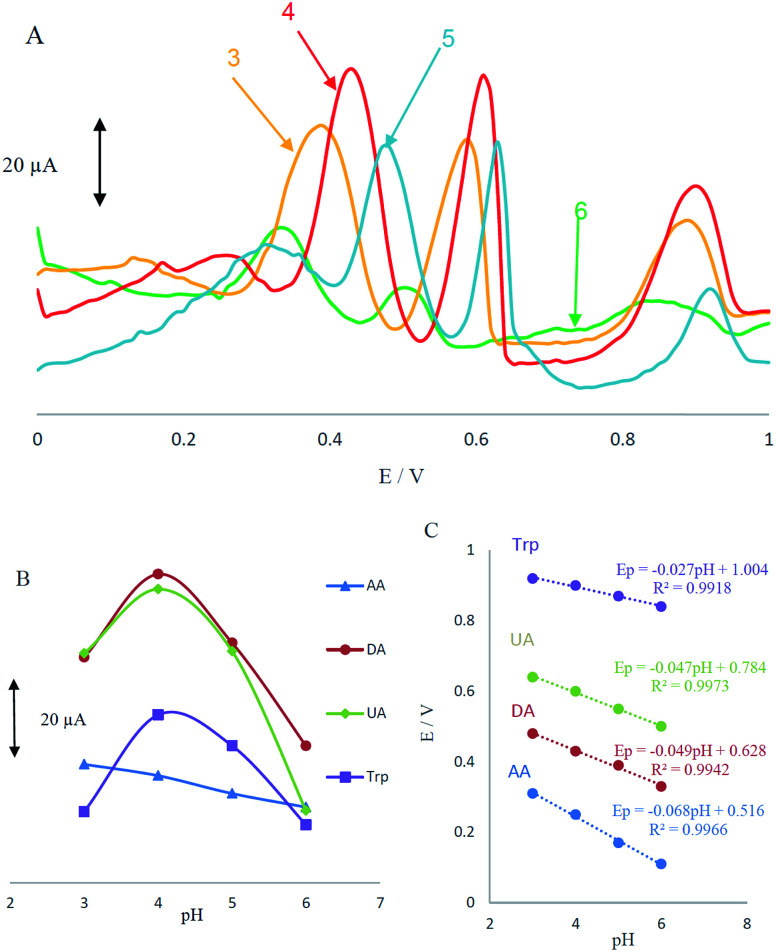
(A) Differential pulse voltammograms of MCCME-BB in a solution containing AA (400 μM), DA (200 μM), UA (200 μM) and Trp (200 μM) in 0.1 M PBS with ranging pH from 3.0 to 6.0; (B) plots of peak potential *versus* pH for four analytes; (C) plots of peak current *versus* pH for four analytes.

Acidity of the electrolyte has a significant influence on the AA, DA, UA and Trp electrooxidation, because protons take part in the electrode reaction. The effect of pH at MCCME-BB signal was carefully investigated using DPV in PBS at pH levels ranging from 3 to 6. The results are shown in Fig. 4A.

Based on the results, the peak current of AA, DA, UA and Trp increased slightly with an increase in the solution pH until it reached 4 and then decreased. Fig. 4B displays that the highest peak current and best peak shape was obtained at pH 4 for the AA, DA, UA and Trp. It was observed that as pH of the medium was gradually increased, anodic peak potentials (*E*_p_) for AA, DA, UA and Trp shifted towards less positive values, showing that protons have taken part in their electrode processes. Out of these, based on Fig. 4B, PBS at pH 4 gave the best response in terms of peak current, peak shape and negative shift; hence it was selected as the optimal pH for further studies. Plot of *E*_p_*vs.* pH for AA, DA, UA and Trp in the working pH range is shown in Fig. 4C. As shown, the *E*_p_ of all compounds have a linear relationship with buffer pH. The observed slopes of 0.068, 0.049 and 0.047 mV pH^−1^ for AA, DA and UA, was close to the anticipated Nernstian value for a two-electron, two-proton transfer in the electrochemical reaction but for Trp (with p*K*_a1_ = 2.46 and p*K*_a2_ = 9.41) the slope is 0.027, which was close to the anticipated Nernstian value for a two-electron, one-proton transfer in electrochemical reaction.^44^

### 3.4 Interference studies

**Fig. 5:**
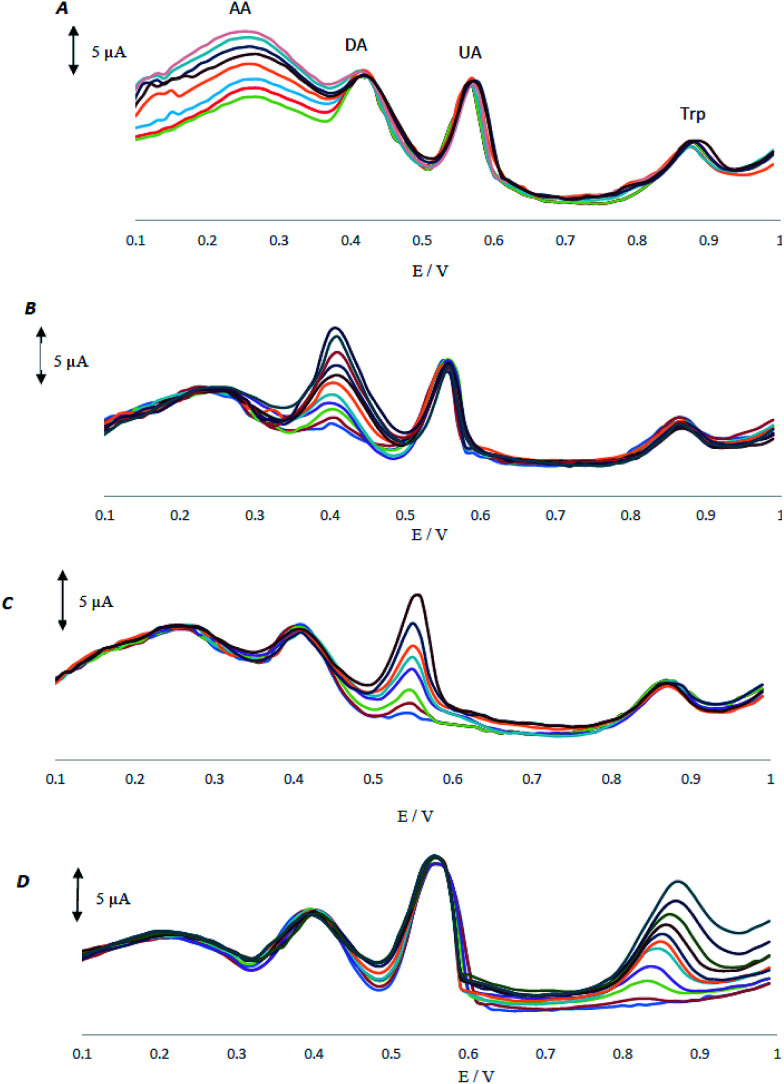
(A) Differential pulse voltammograms of MCCME-BB in 0.1 M PBS (pH 4.0) (A) containing DA (200 μM), UA (100 μM), and Trp (100 μM) and various concentrations of AA (120–450 μM), (B) containing AA (200 μM mM), UA (100 μM), and Trp (100 μM) and various concentrations of DA (8–320 μM), (C) containing AA (200 μM), DA (100 μM), and Trp (100 μM) and various concentrations of UA (4–140 μM), (D) containing AA (200 μM), DA (100 μM), UA (100 μM) and various concentrations of Trp (0–320 μM).

As AA, DA, UA and Trp usually coexist in real samples, it is of great interest to study the interferences between them for the selective detection of each species. In all control experiments, the concentration of one species was changed, while the concentrations of the other species were kept constant. The results are shown in Fig. 5A–D.

As shown in Fig. 5A, peak current of AA increases with an increase in the concentration of AA, while the peak current for the oxidation of DA, UA and Trp remained constant. Fig. 5B–D demonstrate that the voltammetric peaks corresponding to the oxidation of DA, UA and Trp were found to increase linearly in agreement with the increase in their concentration of DA, UA and Trp, whereas the peak current for the oxidation of other three compounds remained constant. The results showed that the peak currents were linearly proportional to the concentrations of AA (or, DA, UA and Trp), while those of the other three analytes did not change; indicating that the oxidation of AA, DA, UA and Trp at MCCME-BB took place independently.

### 3.5 Simultaneous determination of AA, DA, UA and Trp

DPV was performed to investigate the relationship between the peak current and concentration of AA, DA, UA and Trp.

**Fig. 6:**
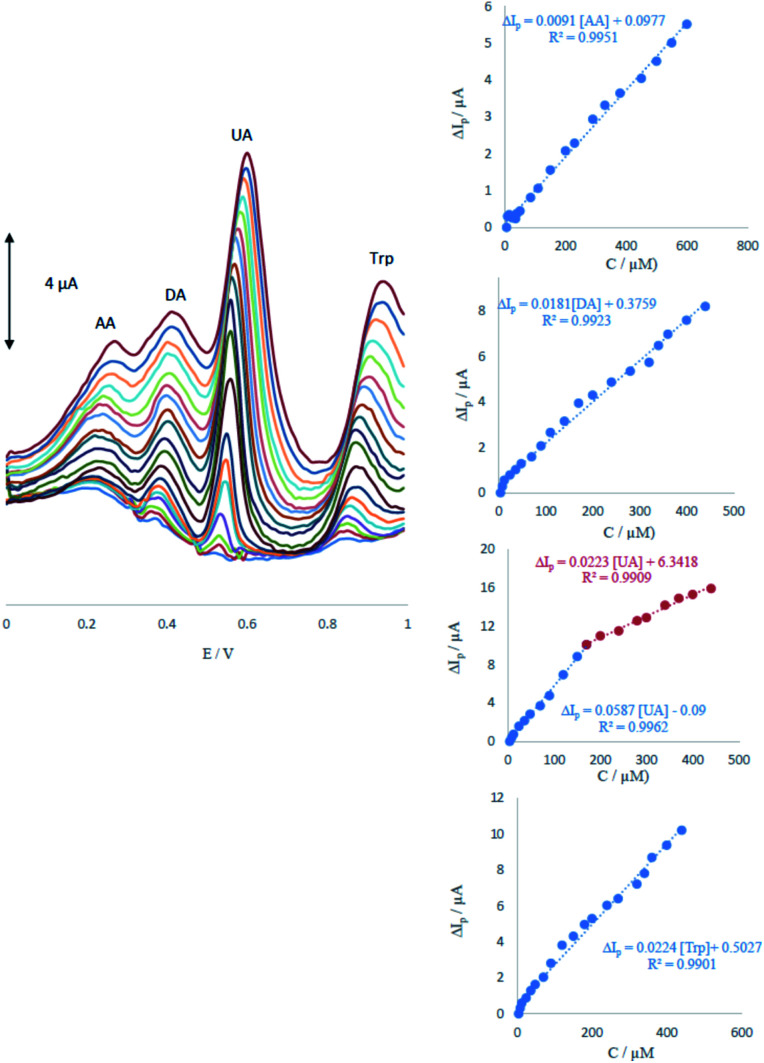
Differential pulse voltammograms of MCCME-BB in 0.1 M PBS (pH 4.0) for the simultaneous detection of four analytes at varying concentrations. Insets display the plots for the concentration dependence of anodic peak current signals with the linear range marked in blue.

As shown in Fig. 6, the DPV curves showed four well-distinguished oxidation peaks. Voltammograms clearly show that the plot of the peak current *versus* AA, DA and Trp concentration is composed of one linear segment but Trp with two segments with different slopes (see insets of Fig. 6). Anodic peak currents of AA, DA, UA and Trp at the surface of ME were linearly dependent on the AA, DA and Trp concentrations with one segment over the range of 6–600, 4–440, 4–440 μM and two segments for UA over the ranges 4–170 and 170–400 μM. The detection limits were determined as 1.65, 0.83, 0.36 and 1.26 μM for AA, DA, UA and Trp, respectively.

### 3.6 Stability and reproducibility

**Fig. 7:**
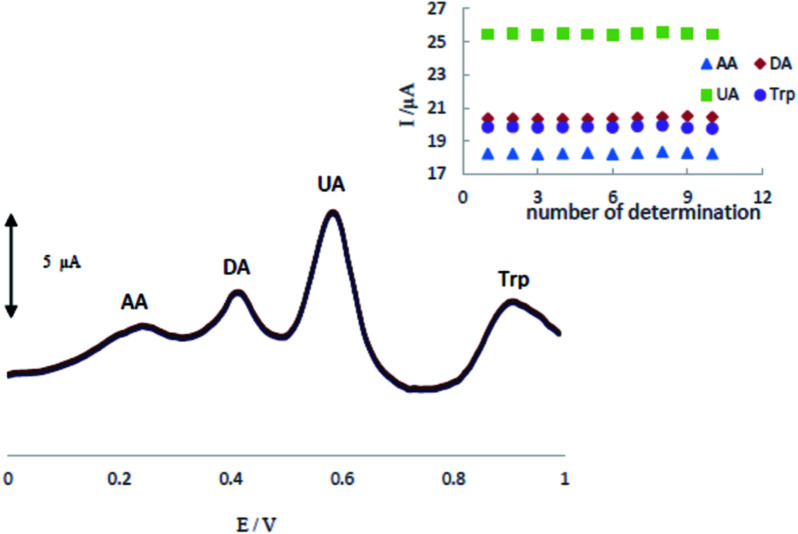
The stability of repetitive measurements (*n* = 10) of DPV response of MCCME-BB using AA (300 μM), DA (200 μM), UA (200 μM), and Trp (200 μM) in 0.1 M PBS (pH 4).

The stability and reproducibility of the MCCME-BB were investigated for ten successive determinations (*n* = 10). Differential pulse voltammograms of a mixture of AA (300 μM), DA (200 μM), UA (200 μM), and Trp (200 μM) in 0.1 M PBS (pH 4.0.) at MCCME-BB are shown in Fig. 7. The relative standard deviation of results were calculated as 0.32%, 0.34%, 0.25% and 0.35% for AA, DA, UA and Trp, respectively. So, the electrochemical signals of analytes on the MCCME-BB electrode had an excellent stability and reproducibility.

### 3.7 Real sample analysis

To evaluate the practical applicability of the proposed modified electrode, the MCCME-BB was examined for the simultaneous determination of AA, DA, UA and Trp in the human urine and blood serum samples.

As shown in Table 1, the samples were diluted with PBS and DPVs were used for the simultaneous determination of AA, DA, UA and Trp using standard addition method. Statistically acceptable recovery values were obtained that indicated the applicability of this modified electrode for highly sensitive detection of these biomolecules in real samples.

**Table 1:** Simultaneous determination of AA, DA, UA and Trp in human urine and blood serum samples (*n* = 3)

Sample	Analyte	Detected (μM)	Added (μM)	Found (μM)	Recovery (%)
Serum 1	AA	—	60	59.1	98.62
	DA	—	60	61.7	107.83
	UA	48.3	60	106.4	98.24
	Trp	—	60	58.7	97.8
Serum 2	AA	—	60	60.8	101.3
	DA	—	60	62.3	103.8
	UA	34.2	60	96.7	102.65
	Trp	—	60	66.1	102.66
Urine 1	AA	—	60	58.5	97.5
	DA	—	60	58.9	98.16
	UA	135.7	60	198.4	101.37
	Trp	—	60	57.9	96.5
Urine 2	AA	—	60	59.3	98.8
	DA	—	60	62.6	104.3
	UA	94.3	60	157.8	102.26
	Trp	—	60	59.6	99.33

## 4. Conclusions

In this report, we are reporting the simultaneous electrochemical determination of AA, DA, UA and Trp for the first time using a MCCME-BB. Compared with the bare electrode, a significant increase of anodic peak current was observed at the MCCME-BB, which clearly demonstrated that the BB could be used as an efficient modifier to enhance the kinetics of the electrochemical process of AA, DA, UA and Trp. Optimization of the experimental conditions yielded a detection limit for AA, DA, UA and Trp of 1.64, 0.82, 0.36 and 1.22 μM, respectively. In addition, the electrochemical sensor was successfully applied for the simultaneous determination of AA, DA, UA and Trp in real samples with promising results for future development of a diagnostic device. The figures of merit such as linear range, limit of detection of the MCCME-BB are compared with other modified electrodes in literature (Table 2). Based on the data in Table 2, the MCCME-BB seems to provide a favourable alternative for the simultaneous determination of AA, DA, UA and Trp in real samples with satisfactory results. A fast and simple fabrication procedure, a wide linear range, high stability and good reproducibility for repeated determinations, suggest that the proposed modified electrode is a potential candidate for simultaneous determination of redox-active biomolecules in real samples.

**Table 2:** Comparison of the proposed modified electrode MCCME-BB with other modified electrodes in literature for the simultaneous determination of AA, DA, UA and Trp

Electrode	Method	Linear response range (μM)				Limit of detection (μM)				Ref.
		AA	DA	UA	Trp	AA	DA	UA	Trp	
RGO-PAMAN-MWCNTs-AuNPs/GCE	DPV	20–1800	10–320	1.0–114	—	6.77	3.33	0.33	—	24
[Ni(phen)_2_]^2+^/SWCNTs/GCE	CV	30–1546	1–780	1–1407	—	12	1	0.76	—	25
Hnp-PtCu^0^/GCE	DPV	200–1000	0.1–1000	5–1000	—	25.01	0.1	2.3	—	26
CTAB-GO/MWCNTs/GCE	DPV	5.0–300	5.0–500	3.060	—	1	1.5	1	—	27
Fe-Meso-PAN	LSV	10–300	10–300	10–300	10–300	6.5	9.8	5.3	5.2	28
AgNPs-rGO/GCE	LSV	10–800	10–800	10–800	10–800	9.6	5.4	8.2	7.5	29
NiCoO_2_/GCE	DPV	0.5–485.4	—	0.5–485.4	0.5–485.4	3.3		5.3	5.7	30
MCCME-BB	DPV	6–600	6–600	6–600	4–440	1.6	0.8	0.36	1.22	This work

## Conflicts of interest

There are no conflicts to declare.

## Supplementary Material
